# Gastric Cancer in the Excluded Stomach 10 Years after Gastric Bypass

**DOI:** 10.1155/2015/468293

**Published:** 2015-07-01

**Authors:** Augusto Tinoco, Lorena F. Gottardi, Eduardo D. Boechat

**Affiliations:** Departamento de Cirurgia Minimamente Invasiva, Hospital São José do Avaí, 28300-000 Itaperuna, RJ, Brazil

## Abstract

According to the Brazilian health authorities, around 2,000 new cases of gastric cancer emerge in Brazil per year (Instituto Nacional de Câncer José Alencar Gomes da Silva, 2014). Indeed, gastric cancer constitutes the second most common cause of cancer-related mortality worldwide and 95% of such malignancies are adenocarcinomas (De Roover et al., 2006, and Clark et al., 2006). Roux-en-Y gastric bypass (RYGB) is a procedure frequently employed in bariatric surgery but restricted access to the excluded stomach means that discovery of gastric lesions is difficult, and diagnosis and treatment may be delayed. We report herein a case of gastric adenocarcinoma in the excluded stomach of a patient submitted to RYGB with the purpose of illustrating the difficulty of diagnosing and treating this rare condition.

## 1. Case Presentation and Management

A 56-year-old white female was brought into the emergency department presenting with severe abdominal pain and distension and reporting flatus expulsion and melenic stools in the previous two days. The patient had been experiencing nausea with cramping abdominal pain in the left flank, but with an absence of vomiting, for a period of two months. Although she had sought medical assistance on numerous occasions during this period, the symptoms had not improved.

Ten years earlier, the patient had presented with class III obesity (BMI 50.8 kg/m^2^) and had received laparoscopic RYGB with concomitant cholecystectomy. Postoperative recovery had been uneventful and the patient subsequently complied with nutritional guidelines. Her BMI diminished to 25 kg/m^2^ after one year and remained unchanged over the following years. The patient admitted smoking, but not alcoholism, during the last 30 years and had presented with vitamin D3 deficiency before and after the bariatric procedure.

**Figure 1:**
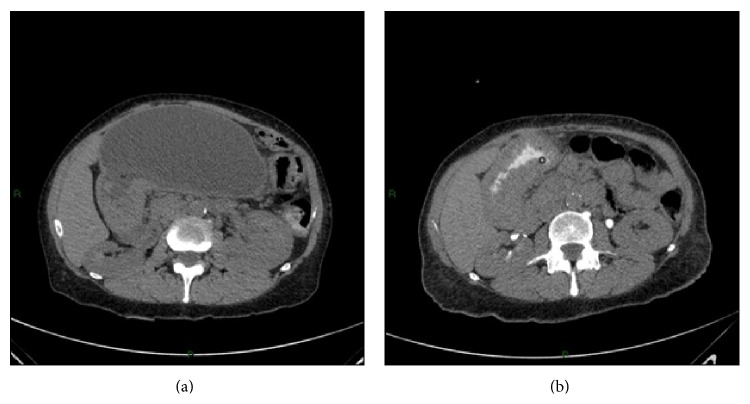
Abdominal computerized tomography showing (a) distension of the excluded stomach and (b) presence of tumor located in the gastric antrum.

Physical examination revealed peristalsis, intense pain on palpation, distended abdomen at the left costal border, and diffuse tympany on percussion. Abdominal computerized tomography (CT) revealed distension of the excluded stomach (Figures 1(a) and 1(b)) and edema in the prepyloric wall. The patient was diagnosed with intestinal obstruction and referred for emergency laparoscopy.

During the course of surgery, distension of the excluded stomach was observed together with a hard lesion in the antropyloric region. Gastrostomy was performed in order to improve the initial clinical conditions and to facilitate diagnosis. Three days later, a further CT scan with intravenous contrast was performed and, by gastrostomy, it was possible to confirm the presence of a mass in the antropyloric region, but with no distant metastasis.

**Figure 2:**
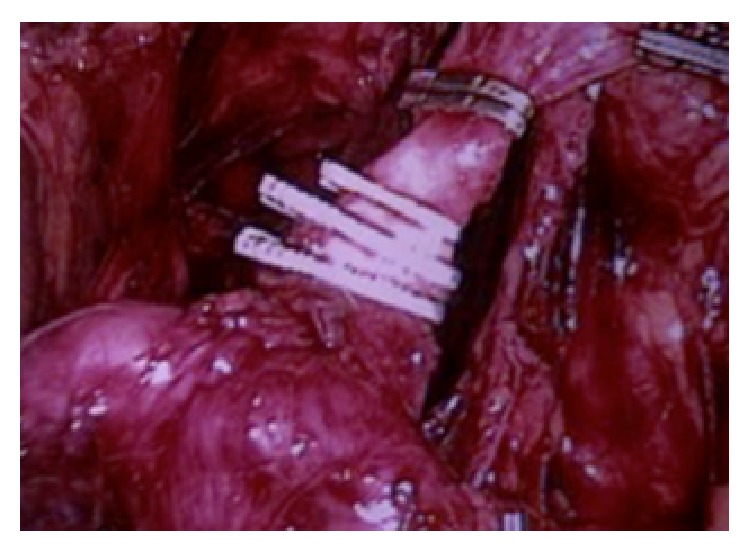
Left gastric artery clipped near the celiac trunk.

Laparoscopic intervention performed seven days later involved total gastrectomy, omentectomy, and D2 lymph node dissection while the primary Roux-en-Y (Figure 2). Postoperative recovery was uneventful. Histopathological analysis confirmed the presence of a moderately differentiated gastric adenocarcinoma, with involvement of the submucosa, and lymphatic and venous tumor emboli. The adenocarcinoma was of the common subtype found in the antropyloric region. The patient was referred to adjuvant chemotherapy since all 26 resected lymph nodes presented metastasis.

The incidence of gastric cancer in the excluded stomach was 1 case (0.03%) in 3047 patients undergoing bariatric surgery between January 1999 and June 2014 at the Surgical Department of the São José do Avaí Hospital.

## 2. Discussion

An important risk factor for the development of gastric cancer is infection of the gastric mucosa by* Helicobacter pylori*, which causes inflammation resulting in premalignant lesions. Other risk factors include a family history of stomach cancer, a diet low in fruit and vegetables, obesity, smoking, and previous gastric surgeries. In the present case, although* H. pylori* infection had been eradicated prior to RYGB, dietary recommendations had been followed, and BMI had been maintained within the normal range after surgery, the patient still presented many of the other risk factors for adenocarcinoma.

Obese individuals exhibit specific alterations in the metabolism of vitamin D and parathyroid hormone (PTH) [1, 2], and gastric bypass commonly reduced levels of vitamin D in the short-term and of PTH in the long-term. In such cases, patients may receive PTH replacement therapy and vitamin D supplementation to prevent possible musculoskeletal disorders resulting from the malabsorption of dietary calcium. It has been shown that vitamin D deficiency is strongly correlated with breast, ovarian, colorectal, prostate, and esophageal cancers as well as melanoma, but the association with gastric adenocarcinoma is still controversial [3]. It is noteworthy that the present patient presented with both pre- and postoperative vitamin D deficiency.

A number of hypotheses seek to explain the development of cancer of the excluded stomach. Moreels et al. [4] suggested that exposure of the stomach to pancreaticobiliary reflux in the presence of bile forms part of the etiopathogenesis of gastric cancer. This proposal was supported by Park et al. [5] who showed that superficial gastritis is common in the proximal and distal stomach, thereby adding to existing risk factors. According to other researchers, the stomach suffers following prolonged contact with stagnant bile and this may lead to chemical carcinogenesis [6]. Additionally De Roover et al. [7] stated that increased pressure and chronic irritation in the gastric pouch may be a carcinogenic risk.

The risk of cancer in the distal excluded stomach is unknown since few studies have focused on the subject and diagnosis is complicated. Moreover, most cases are diagnosed at advanced stages of the disease, despite nonspecific history of epigastric pain, nausea, and vomiting for a considerable period before diagnosis, and this has contributed to poor prognosis and high mortality [7]. Given that endoscopic access to the bypassed stomach for diagnostic purposes is difficult, an alternative approach may be to use a pediatric colonoscope or double-balloon enteroscope to enter the biliopancreatic loop of the Roux-en-Y [8].

The incidence of gastrointestinal stromal tumor in morbidly obese individuals submitted to RYGB is generally low (around 0.8%) but much higher than that of the general population (0.0006–0.0015%) [7]. However, most studies have shown that obese patients who undergo RYGB present a reduced risk of gastric tumor when compared with their nontreated counterparts [9]. In our department at the São José do Avaí Hospital, we have encountered only one case of gastric cancer out of 3,043 patients (0.03%) submitted to bariatric surgery between January 1999 and June 2014. Additionally, only four cases of adenocarcinoma have been reported for patients who had received vertical banded gastroplasty (an alternative restrictive procedure), and these events occurred 2 to 15 years after surgery [7, 10–12]. The emergence of cancer after RYGB can be similarly protracted, although Harper et al. [13] reported the case of a 45-year-old woman who had disseminated gastric cancer involving the excluded stomach one year after RYGB.

Surgical resection to remove the primary lesion and to clear the proximal and distal margins is the only option for patients with gastric adenocarcinoma. Furthermore, resection of the greater omentum and D2 lymphadenectomy is advisable in order to allow appropriate staging and to improve long-term survival [14, 15]. These procedures were employed in the case of the patient described herein who has survived for 1.6 years following diagnosis of adenocarcinoma.

## 3. Conclusion

The incidence of gastric cancer in the excluded stomach was 1 case (0.03%).

The risk of cancer in the distal excluded stomach is unknown since few studies have focused on the subject and diagnosis is complicated. Moreover, most cases are diagnosed at advanced stages of the disease, despite nonspecific history of epigastric pain, nausea, and vomiting for a considerable period before diagnosis, and this has contributed to poor prognosis and high mortality. Radical gastrectomy with lymphadenectomy is the best option to allow appropriate staging and to improve long-term survival for patients with gastric adenocarcinoma.
